# An explorative study comparing skin surface lipids in the West Highland white terrier dog with and without atopic dermatitis

**DOI:** 10.1080/01652176.2022.2028033

**Published:** 2022-01-19

**Authors:** Helen L. Orbell, Nick J. Cave, Katharina Parry, Craig E. Griffin

**Affiliations:** a Animal Dermatology Clinic, Palmerston North, New Zealand; b School of Veterinary Science, Palmerston North, New Zealand; c AgResearch, Palmerston North, New Zealand; d Animal Dermatology Clinic, San Diego, CA, USA

**Keywords:** Canine, dog, West Highland White terrier, atopy, lipidome, sebaceous, cutaneous, epidermis, stratum corneum, mass spectrometry

## Abstract

**Background:**

The skin barrier is important in the pathogenesis of atopic dermatitis and stratum corneum lipids have a critical role. Skin surface lipids have been largely overlooked but also contribute to barrier function. An untargeted approach was used to compare the skin surface lipids from atopic and non-atopic West Highland White terrier dogs (WHWT).

**Objectives:**

The primary hypothesis was that a difference in the lipidome would exist. The secondary hypothesis was that affected and unaffected skin lipids would differ.

**Animals and methods:**

This prospective, cross-sectional, case-controlled study included thirty-nine privately owned WHWTs. Dogs were assigned to one of four disease status groups based on strict criteria. Samples for lipid analysis were collected from the skin surface of unaffected and affected sites. Lipid analysis was by untargeted liquid chromatography/mass spectrometry and utilised lipid identification software packages. Principle component analysis (PCA) and partial least-squares discriminant analysis (sPLS-DA) statistical methods analysed the association between the relative lipid abundance and disease status and affected and unaffected skin.

**Results:**

Samples for lipid analysis found 421 lipid soluble features of which ten lipids were positively identified. Statistical analysis could not distinguish between non-atopic and atopic dogs but did reveal a statistically significant difference in the lipid profiles from affected and non-affected skin irrespective of disease status.

**Conclusions:**

A large array of unidentified lipids from the skin surface were found with a difference between affected and unaffected skin unrelated to disease status. Investigation into the lipidome of the skin surface is an emerging area of research with clinical and therapeutic applications.

## 1. Introduction

The pathogenesis of atopic dermatitis (AD) is complex and multifactorial. There is evidence an impaired skin barrier plays a role in the pathogenesis (Marsella 2014) which may include stratum corneum (SC) lipid abnormalities (Inman et al. 2001; Stahl et al. 2001; Olivry 2011; van Smeden et al. 2011; Miller et al. 2013; Nishifuji 2014). The skin barrier is thought to be provided predominantly by the outermost layer of the epidermis, the SC (Olivry 2011; Miller et al. 2013). The SC is composed of layers of corneocytes that are surrounded by an intercellular lamellar lipid matrix mainly composed of ceramides, cholesterol and free fatty acids (Nishifuji 2014). In addition to the intercellular lipids, the superficial surface of the SC is also coated in lipids. The skin surface lipids are species specific and are predominantly derived from the sebaceous glands with a small proportion derived from the corneocytes of the SC (Pappas 2009). In humans, it is the combination of lipids within and upon the SC that provide a hydrophobic barrier to prevent evaporative drying of the epidermis and restrict access of environmental allergens to the cutaneous immune system (Pappas 2009). In previous studies of the cutaneous lipids, targeted approaches have focused on specific lipid classes within the SC, such as ceramides, cholesterol and free fatty acids (Reiter et al. 2009; Shimada et al. 2009; Yoon et al. 2011; Popa et al. 2012; Chermprapai et al. 2018). The skin surface lipids have been largely disregarded because it has been suggested they do not play a major role in skin barrier function (Kligman 1963). Consequently, very little is known about the skin surface lipids in relation to their composition and variability which may have resulted in an incomplete picture of skin lipid composition and abnormalities in disease. There are several studies indicating that surface lipids do in fact have an important role in skin barrier function and hydration of the SC (Pappas 2009; Dahlhoff et al. 2016; Lovászi et al. 2017). In asebia SCD1 knockout mice that have profound sebaceous gland hypoplasia, there is a marked decrease in SC hydration resulting in epidermal hyperplasia, microscopic evidence of inflammation and excess scale (Fluhr et al. 2003). The surface lipids, and in particular the sebaceous lipids, are also generating renewed interest as it emerges from human *in-vitro* studies that, in addition to their role in barrier function, they may also have antimicrobial effects, they have pro- and anti-inflammatory properties, and have a role in immune modulation (Lovászi et al. 2017; Mattii et al. 2018). To progress our understanding of the complex composition and role of skin lipids it is necessary to use an unbiased global (untargeted) approach to determine the relative abundances of the different lipid classes and to explore if any of these vary when disease occurs. To the authors’ knowledge, there has not previously been an untargeted study into the composition of skin lipids, or an untargeted comparison of lipids in atopic and non-atopic skin in the dog.

The primary aim of this study was to investigate the skin surface lipids in healthy non-atopic and atopic dogs, utilising liquid chromatography-mass spectrometry (LC-MS). This study was conducted using a group of privately-owned West Highland White terrier dogs (WHWT). The WHWT is a breed that is recognised to be predisposed to developing AD (Jaeger et al. 2010; Salzmann et al. 2011; Favrot et al. 2020) causing concern to owners whilst at the same time providing opportunities for research into the pathogenesis of canine AD.

The primary hypothesis of the study was that there would be a difference in the skin lipid composition between WHWTs with and without AD. The secondary hypothesis was that there would be a difference in the lipid composition between affected and unaffected skin.

## 2. Materials and methods

The study was carried out as a prospective, cross-sectional, case-controlled study. Dogs were privately-owned and client written consent was obtained. The study was approved by the Massey University Animal Ethics Committee (Protocol 17/104).

### 2.1. Animals

Forty-three WHWTs were initially enrolled in the study with an age range of four months to 15 years. Dogs were recruited from the West Highland White & Scottish Terrier Club (38 dogs) and from the Animal Dermatology Clinic (five dogs). A list of 50 dog owners who were members of the West Highland White & Scottish Terrier Club was provided by the club secretary. Forty-four WHWT club members were contacted based on their geographical location and there were 22 responses from owners willing to allow their dog(s) to be a part of the study. Several owners had more than one of their dogs included in the study. Owners were advised not to bathe the dogs for at least seven days prior to day of examination. All other current medications including topical, systemic medications and immunotherapy were permitted and their use recorded.

### 2.2. Designation to disease status group

Each dog was allocated to one of four skin disease status categories: 1. Apparently Healthy/Not Atopic (HNA) 2. Atopic dermatitis (AD) 3. Likely Atopic dermatitis (LAD) 4. Other skin disease/Not Atopic (ONA). Allocation to one of these 4 categories was determined based on strict criteria which are outlined in Table 1. The emphasis was to ensure that only healthy and non-atopic dogs were included in the HNA group and only atopic dogs were included in the AD group. The criteria included the presence of pruritic behaviour based on the answers given in an owner survey, a validated owner-assessed pruritus visual analogue scale (PVAS) score, Canine Atopic Dermatitis and Extent Severity Index 04 (CADESI-04) score and calculation of the number of Favrot’s criteria that were met. Favrot's criteria are a validated set of clinical features which are associated with canine AD (Favrot et al. 2010). They were developed to assist in the diagnosis of canine AD but not to be used alone for diagnosis. There are 2 sets of Favrot's criteria which serve different purposes. Set 1 is more subjective and used for clinical studies. Set 2 was applied because it was the most applicable when evaluating for the probability of a diagnosis of AD. If 6/7 criteria are met for Favrot’s Set 2 then the highest possible specificity for AD can be achieved (specificity 93.7%, sensitivity 42%). If 5/7 criteria are fulfilled the specificity is 83% and sensitivity increases to 77.2%. A comprehensive 60-question survey using the online platform SurveyMonkey® was completed by owners (Additional file 1). The survey questions were modelled on those used in a previous study which established levels of pruritic behaviours in apparently healthy dogs without skin disease (Stetina et al. 2015). Behaviours which were considered above the normal level of pruritus included paw licking/chewing daily or multiple times a day, face or muzzle rubbing multiple times a day and head shaking multiple times a day. A complete dermatological examination was performed by the primary investigator and skin lesions were recorded using the validated CADESI-04 scoring system (Olivry et al. 2014). The primary investigator obtained a PVAS score (Hill et al. 2007; Rybnícek et al. 2009) at the time of examination which is a validated owner-assessment of the level of pruritus. A PVAS score of 1.9 or less is considered a normal score (Rybnícek et al. 2009; Stetina et al. 2015) for a healthy dog or a dog with AD in remission.

**Table 1.** Dogs were assigned to 1 of 4 disease status groups based on the combined criteria outlined for pruritic behaviour, owner assessed pruritus score (PVAS), Favrot’s criteria set 2, CADESI-04 score and relevant history and physical examination findings.

Disease status Criteria	Apparently Healthy/Not atopic (HNA)	Atopic dermatitis (AD)	Likely atopic (LAD)	Other skin disease-Not atopic (ONA)
PVAS	<1.9	≥1.9	≥1.9	<1.9
CADESI-04	<10	≥10	≥10	≥10
Pruritic behaviour	No	Yes	Yes	No
Favrot’s criteria Set 2	N/A	≥5	≤4	N/A
Relevant history and dermatological examination	No history of any skin or ear disease, no skin or ear medications	History and DE consistent with AD. Medications for pruritus, skin and ear infections, otitis	History and DE consistent with AD. Medications for pruritus, skin and ear infections, otitis	History and DE not consistent with AD.No medications for pruritus

DE = dermatological examination. N/A = not applicable because dog not pruritic.

The combination of these criteria was applied to designate each dog to a disease status group. When identifying pruritic individuals, more emphasis was placed on abnormal levels of pruritic behaviours identified in the survey than the PVAS score. A PVAS of up to 1.9 was the highest score allowed in the HNA group but only if there was no abnormal pruritic behaviour revealed in the survey.

### 2.3. Sample site selection

Sample sites were kept as consistent as possible between dogs. The medial aspect of the hindleg (MHL) was chosen as the preferred site because it is relatively sparsely haired in the WHWT and provided a large enough area for the samples to be collected comfortably. Samples were collected from the MHL in all dogs. If the MHL was affected, then an unaffected site was chosen in order of preference, where possible, from the ventral abdomen, lateral chest, axilla or dorsum. Affected skin was defined as skin exhibiting erythema with or without other primary or secondary lesions including alopecia, papules, pustules, epidermal collarettes, excoriations, crusts, lichenification, hyperpigmentation and seborrhoea. If the skin to be sampled was in a haired area, then the hair was clipped from the site using electric clippers taking care not to disturb the skin surface.

### 2.4. Sample collection

To collect the lipid sample, a flexible plastic board (12 × 14 cm) with a central rectangular hole (2 × 4 cm) was pressed to the surface of the skin. A cotton-tipped applicator whetted with acetone and n-hexane (1:1 v/v) was firmly rolled over the exposed area of skin to cover the entire surface once for 24.5 +/− 4.5 seconds. The cotton-tipped applicator was then placed back into the solvent into a screw-top plastic tube and stored at −20 degrees Celsius until analysis.

### 2.5. Lipid extraction

All individual samples were extracted with Folch solution (CHCl_3_: MeOH: H_2_O, v/v/v, 65:33:2), containing phosphoethanolamine (16:0 D31-18:1, PE) as the internal standard (Folch et al. 1957). Then 2 mL of solvent was added to each sample, vortexed for 60 seconds, and the extract dried down using a SpeedVac concentrator at 30 °C. The residue was re-dissolved in 200 µL Folch solution, vortexed for 60 seconds, centrifuged at 14,000 rpm for 3 minutes and transferred into a sample vial to be directly used for analysis.

### 2.6. Lipid analysis

Mass spectrometric analysis was carried out on a Q-Exactive Mass Spectrometer (Thermo Fisher Scientific, San Jose, CA, USA) using positive and negative electrospray ionisation and collection of MS2 spectra from m/z 200–2000. Samples were injected onto an Acquity CSH C18 column (1.7 µm, 2.1 mm × 100 mm; Waters, Milford, MA, USA). Lipids eluted over a 15 min gradient from 85% solvent A (60% Acetonitrile, 0.1% formic acid, 10 mM NH_4_COOH in Milli-Q water) to 99% solvent B (90% Isopropanol, 10% Acetonitrile, 0.1% formic acid, 10 mM NH_4_COOH). The MS1 data (used for quantification) was collected at 70,000 mass resolution while the MS2 data was collected at 35,000 resolutions. Lipid concentrations were calculated by using the known amount of standard in each sample and calculating the concentrations of peaks detected relative to the amount of standard added. While not accounting for the relative differences of ionisation efficiency between the different lipids detected, it does provide an estimate of the relative abundance for comparison between the different samples. The average coefficient of variation for standard detection was 19%. The LC-MS was performed under contract at AgResearch, Palmerston North, New Zealand. Data files were converted to mzXML format using the file converter ProteoWizard. Non-targeted peak detection was performed using the R package XCMS. The resulting peak area matrix data files were de-isotoped using a custom script written in R version 2.15.0 (R Development Core Team, 2012). The data was analysed using the lipid identification software package LipidSearch (Thermo Fisher Scientific, San Jose, CA, USA). A further number were tentatively identified in positive ion mode using Lipidblast. Data are reported as relative abundances (mol%), whereby 100% corresponds to the total amount of lipids present.

### 2.7. Statistical analysis

Raw data were the relative abundances of each lipid moiety. Absolute concentrations were not used as the total amount of lipid extracted from each swab was not determined. The association between relative lipid abundance of all apparently unique moieties and disease status, and the binary classification of ‘affected’ and ‘unaffected’ skin sites was explored using principle component analysis (PCA), and partial least-squares discriminant analysis (sPLS-DA). PCA was used to provide an unconstrained visualisation of the lipid variation between samples. sPLS-DA is an effective tool for determining which quantitative variables are informative in a discriminant analysis. The relative abundances of lipid moieties were used to discriminate disease status and the most useful lipids for predicting disease status were extracted. All analysis and plotting were performed using the prcomp and sPLS-DA functions of the statistical software R (Rohart et al. 2017) (R, version 3.6.0, The R Foundation for Statistical Computing, Vienna, Austria). Prior to analysis, correlation matrices were constructed, and moieties with very high correlations (Pearson’s coefficient >98%) were assumed to represent identical compounds and the more abundant was retained for analysis.

## 3. Results

### 3.1. Animals

A total of 43 WHWTs were initially considered for inclusion ranging in age from four months to 15 years. Four dogs were excluded due to known systemic disease that may have altered their skin lipids. Specifically, two dogs died soon after examination from unrelated causes and were presumed to have been suffering from serious disease. Another two dogs were excluded as they had known systemic diseases—one dog had hyperadrenocorticism and the other diabetes mellitus. Therefore, there were 39 dogs included in the lipid analysis phase.

### 3.2. Dogs in disease status groups

Table 1 summarises the disease status groups for all dogs based on the combined criteria and Table 2 represents the signalment and the disease status group for each dog as well as their individual owner-assessed PVAS, CADESI-04 score and Favrot’s criteria Set 2 score. The information regarding pruritic behaviours obtained from the survey helped identify pruritic dogs (Stetina et al. 2015) even when the PVAS score was normal (Dogs 25, 26, 27 and 28). Favrot’s criteria (Favrot et al. 2010) were strictly applied and only pruritic dogs with compatible clinical history and a Favrot’s score of 5 or 6 were assigned to the AD group. Seven dogs (17.9%, average CADESI 5.7, average age 68 months) were classified as apparently healthy/not atopic (HNA). There were 20 dogs (51.3%, average CADESI 30.95, average age 92 months) classified as Atopic (AD) and five dogs classified as Likely atopic (LAD) (12.8%, average CADESI 36.2, average age 108 months + dog 28 age unknown). There were seven dogs (17.9%, average CADESI 14.28, average age 68 months) that were classified as having other skin disease/Not atopic (ONA). The skin sites sampled are presented in Additional file 2. Three dogs classified as HNA had a single affected site (Dogs 3, 4, 7) consistent with minor focal injury.

**Table 2.** Dogs included in the study with their signalment and assignment to disease status group.

Group	Dog #		Gender			Age months				PVAS		CADESI-04		Pruritus		Compatible Hx/Medication		Favrot	
**Apparently healthy/Not atopic (HNA)**																			
	1^a^		F			9				0		4		No		None		N/A	
	2^a^		FN			72				0		6		No		None		N/A	
	3		MN			15				0		2		No		None		N/A	
	4		FN			42				0		7		No		None		N/A	
	5^a^		MN			120				1.6		5		No		None		3	
	6^a^		FN			106				0		8		No		None		N/A	
	7		MN			111				1.7		8		No		None		3	
**Atopic (AD)**																			
	8		FN			122				6.4		74		Yes		Yes		6	
	9		MN			41				8		44		Yes		Yes		6	
	10		MN			66				5.2		88		Yes		Yes		5	
	11		FN			60				6.4		42		Yes		Yes		5	
	12		MN			42				4.4		17		Yes		Yes		5	
	13		MN			40				6.5		53		Yes		Yes		6	
	14		FN			181				3.6		37		Yes		Yes		5	
	15		MN			149				3.5		35		Yes		Yes		5	
	16		F			44				3.2		33		Yes		Yes		6	
	17		FN			182				3.8		21		Yes		Yes		5	
	18		MN			144				7.5		34		Yes		Yes		5	
	19		FN			80				2.4		10		Yes		Yes		5	
	20		FN			65				1.8		12		Yes		Yes		5	
	21		MN			164				3.3		14		Yes		Yes		5	
	22		FN			68				1.9		36		Yes		Yes		5	
	23		F			9				3.5		10		Yes		Yes		5	
	24		FN			152				3.5		17		Yes		Yes		5	
	25		F			4				0		7		Yes		Yes		6	
	26		MN			129				0		10		Yes		Yes		5	
	27		MN			101				0		25		Yes		Yes		5	
**Likely atopic (LAD)**																			
	28		F			–				0		74		Yes		Yes		4	
	29		FN			125				3.5		15		Yes		Yes		4	
	30		MN			101				7		24		Yes		Yes		4	
	31		FN			187				1.5		19		Yes		Yes		4	
	32		F			18				3.5		49		yes		Yes		4	
**Other disease/Not atopic (ONA)**																			
	33		M			6				0		13		No		No		N/A	
	34		M			7				0		11		No		No		N/A	
	35		FN			118				0		27		No		Yes		N/A	
	36		F			101				0		21		No		No		N/A	
	37		M			38				0		9		No		No		N/A	
	38^a^		FN			112				0		10		No		No		N/A	
	39^a^		FN			40				0		9		No		No		N/A	

F = female entire, FN = female neutered, M = male entire, MN = male neutered. N/A = Favrot score not applicable because dog was not pruritic.

^a^Dogs with an unpaired sample.

### 3.3. Lipid identification

**Figure 1.**
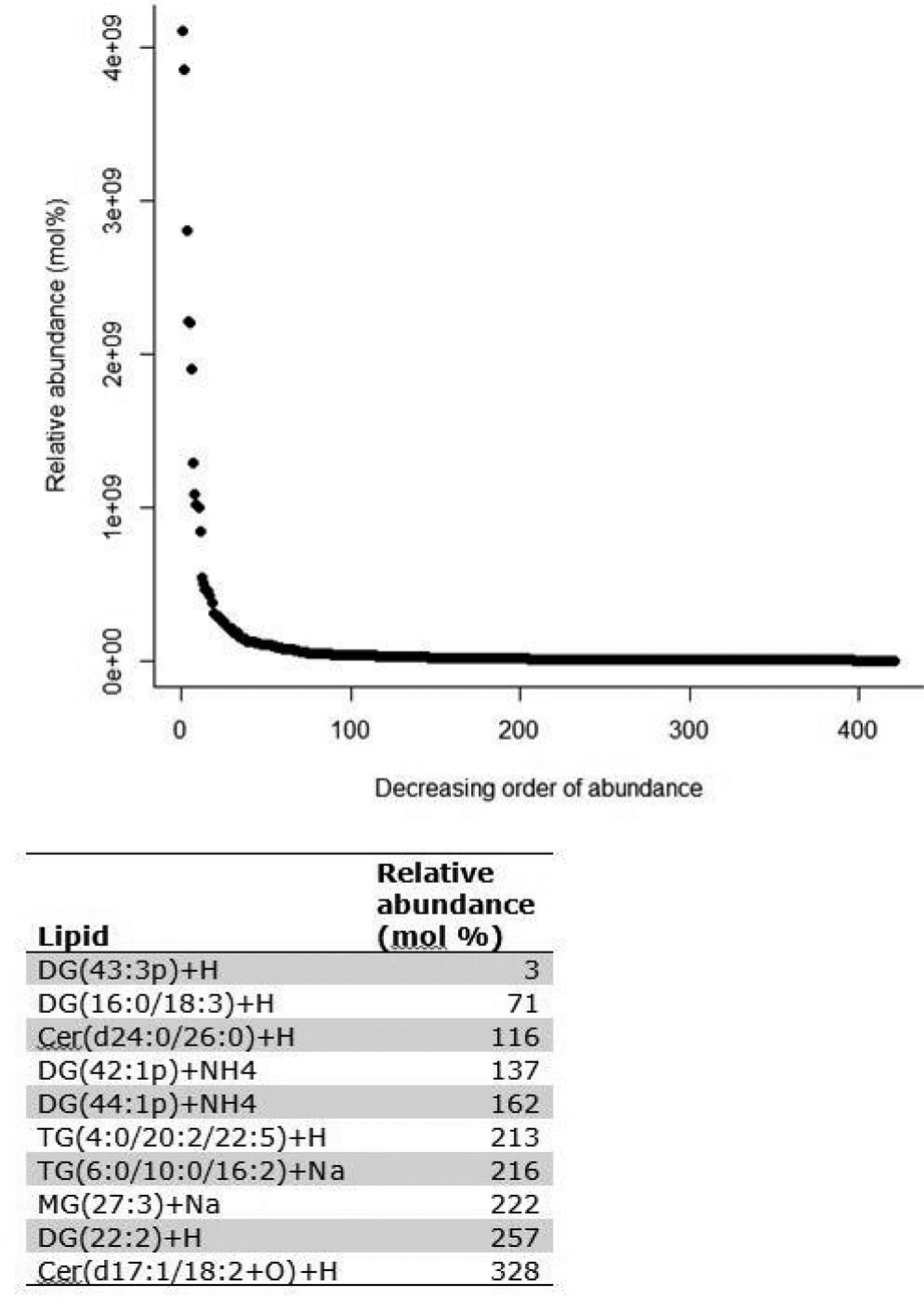
Relative abundance (mol %) of the positively identified skin lipids found on the skin of 39 WHWT dogs.

**Figure 2.**
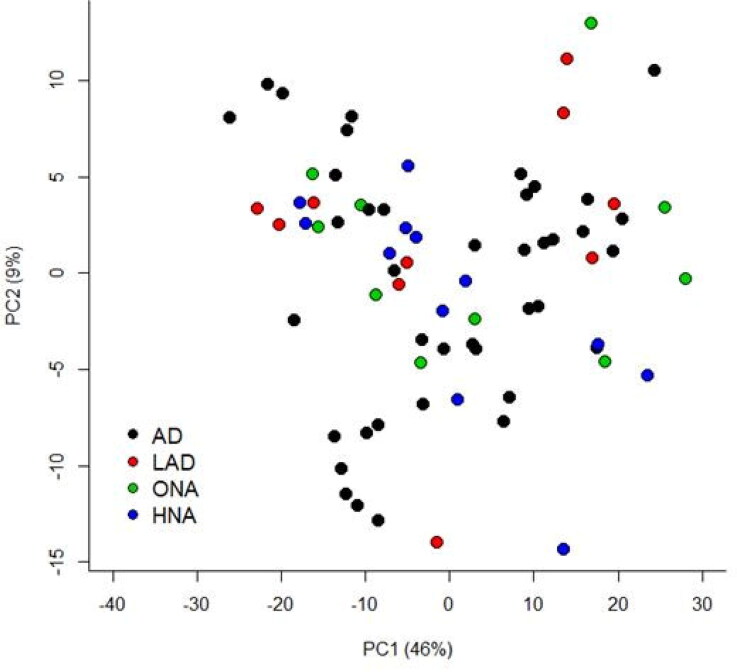
Principal component analysis (PCA) comparing feature profiles of dogs in all disease categories. Both unaffected and affected samples are included in the analysis. Legend describes the disease status groups: AD = atopic dermatitis, LAD = likely atopic dermatitis, ONA = other skin disease, non-atopic, HNA = apparently healthy, non-atopic.

**Figure 3.**
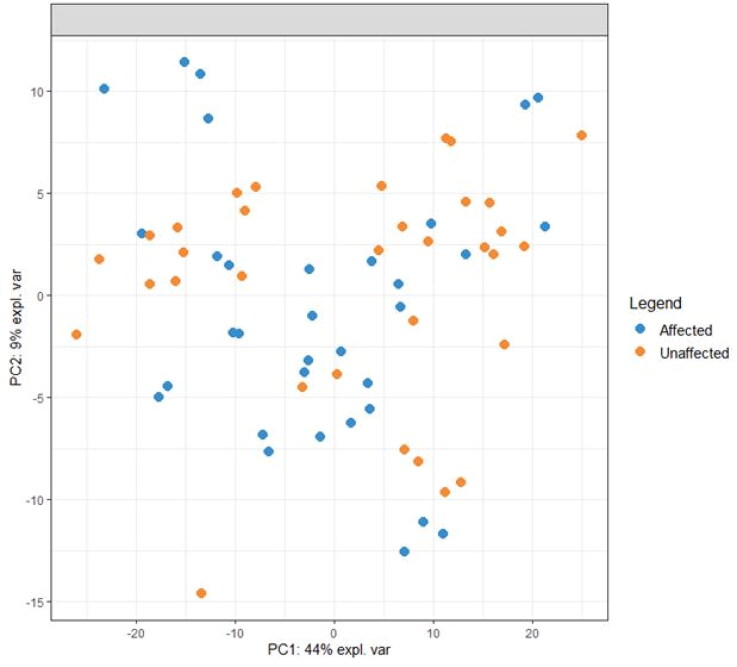
PCA of lipid profiles from Affected and Unaffected sites from all dogs that had paired samples collected. Not represented in this plot are 6 dogs that did not have a paired sample collected (4 HNA dogs and 2 ONA dogs).

**Figure 4.**
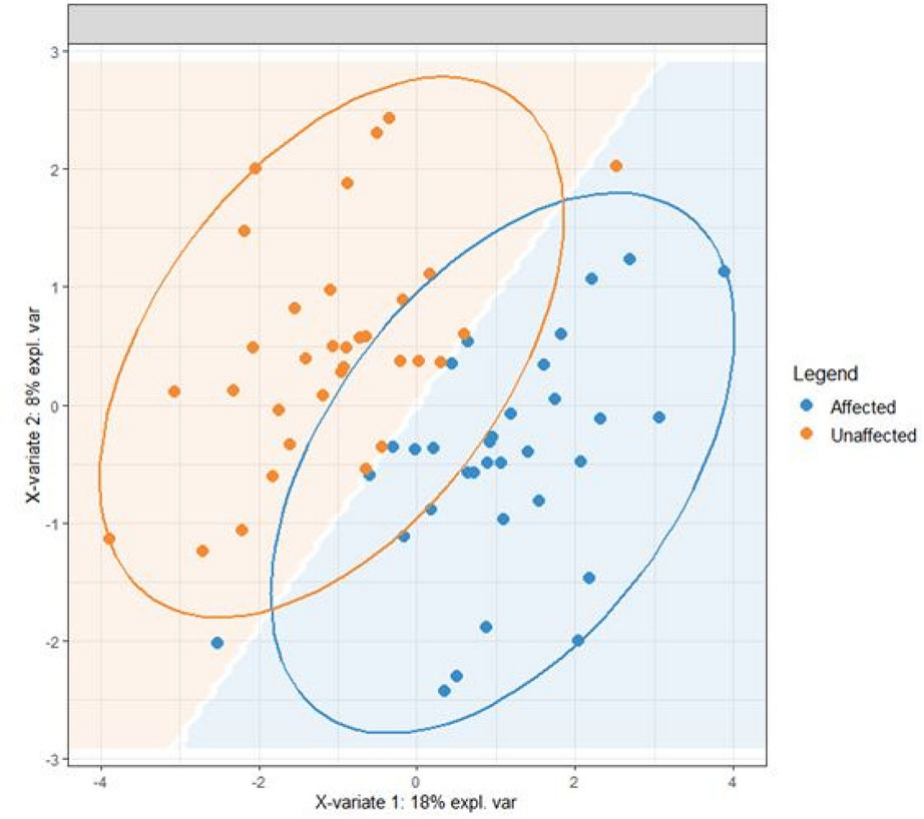
A reduced-space plot of the discriminant axes showing a clear distinction between samples from affected and unaffected sites.

**Figure 5.**
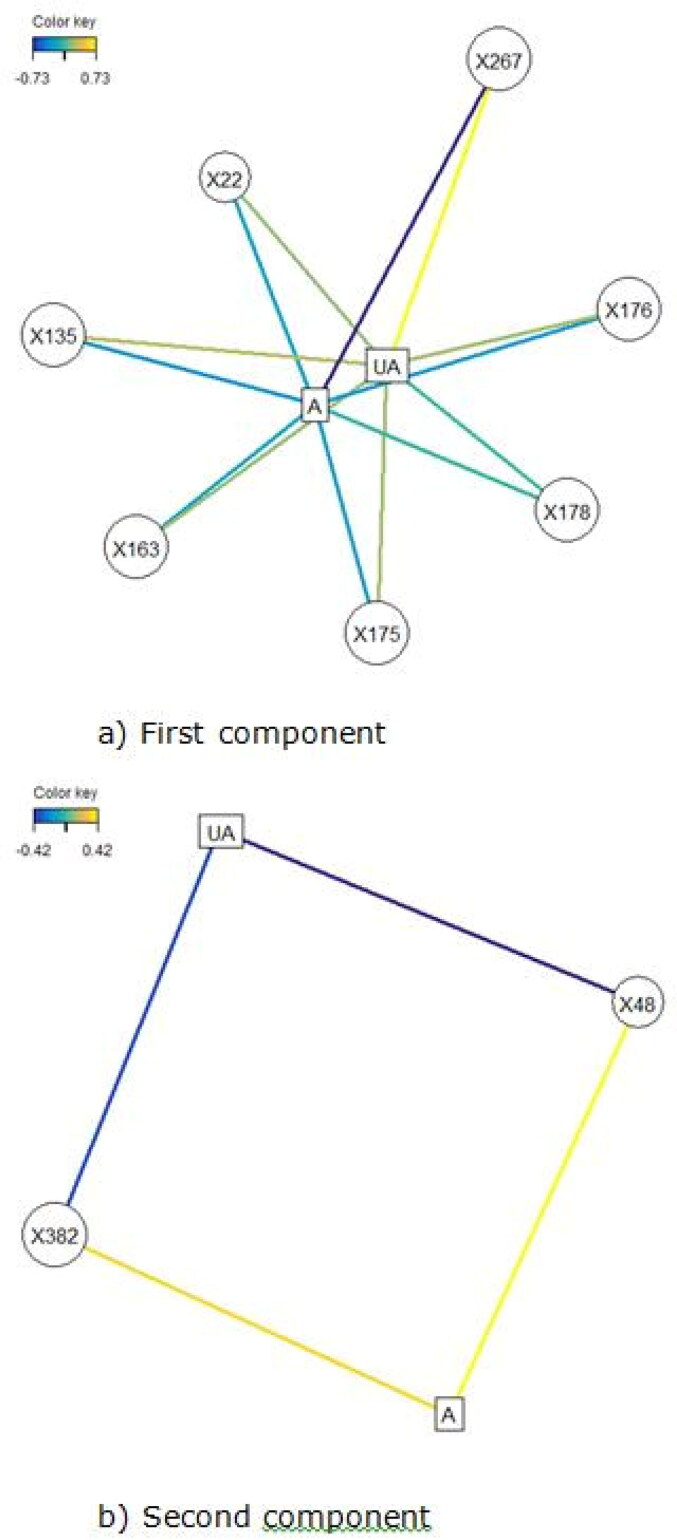
Association network plots for the (a) First component and (b) Second component depict the lipids most likely to be associated with a site being affected or unaffected (A = affected, UA = unaffected). (a) In the first component, positive values are associated with a site likely to be affected so a site high in the lipids shown is likely to be unaffected. Lipid X267 is positively associated with a site being unaffected and is negatively associated with a site being affected. The same is true to a lesser degree for all the lipids shown, with the relationship being weakest for X22. (b) In the second component, positive values were associated with a site likely to be unaffected, therefore sites high in those lipids are more likely to be affected. X48 is positively associated with a site being affected and negatively associated with a site bring unaffected. Lipid X382 is also associated with being affected, but the relationship is weaker.

A total of 421 lipids (mass/retention time lipid soluble features) were present in the skin samples from the 39 dogs. After removal of highly correlated (>0.99 correlation) lipids the total was 392. There were 10 lipids positively identified with perfect alignment using the lipid identification software package LipidSearch (Table 3). These 10 lipids were not the lipids in the highest abundance but were the only fully identified lipids found. There were a further 26 lipids that had a partial match on one of the databases (LipidSearch or LipidBlast) (Table 3). Another 166 lipids were not positively identified but were close to matching on one of the databases. The remaining 217 lipids could not be identified on either of the lipid databases utilised. The relative abundance of the lipids found is depicted in Figure 1 and the associated table. The 10 positively identified lipids ranged from the third most abundant (DG (43:3p) +H) to the 328th most abundant (Cer (d17:1/18:2 + O) +H). PCA was applied to the complete list of lipids from all sites for all 39 dogs. PCA did not reveal any patterns to the profiles when grouped according to disease status (Figure 2) or affected/unaffected site status (Figure 3). There was a large variation between individual dogs, irrespective of disease status, and it was considered that any potential difference according to disease or affected/unaffected appearance might be obscured by that variation. In addition, the PCA did not account for the paired nature of the samples (affected and unaffected sites). Paired samples were available for all AD and LAD dogs, 4/6 of the ONA dogs and 3/7 of the HNA dogs. In order to account for the greater variation between individuals than between samples, the statistical modelling method sPLS-DA was applied to the paired data. A reduced-space plot of the discriminant axes showed a clear distinction between samples from affected and unaffected sites (Figure 4). Association network plots (Figure 5) identify the most discriminatory features which were the lipid X267 in the 1st component and LSF X48 in the 2nd component.

**Table 3.** Positively identified lipids and partially identified lipids present in the skin samples from the 39 dogs with LC-MS using the lipid databases LipidBlast and LipidSearch.

Lipids positively identified	Lipids partially identified
MG(27:3)+Na DG(22:2)+H DG(16:0/18:3)+H DG(43:3p)+H DG(42:1p)+NH4 DG(44:1p)+NH4 TG(6:0/10:0/16:2)+Na TG(4:0/20:2/22:5)+H Cer(d17:1/18:2 + O)+H Cer(d24:0/26:0)+H	MG 20:3; [M + NH4]+ MG 22:6; [M + NH4]+ MG 23:0; [M + NH4]+ DG 26:2; [M + NH4]+ SM 18:0; [M-H + Na]+ SM 32:2; [M-H + Na]+ LPE 22:0; [M + H]+ CerP 29:0; [M + H]+ CerP 29:1; [M + H]+ CerP 41:2; [M + H]+ CerP 42:5; [M + H]+ PA 25:2; [M + Na2-H]+ PA 32:6; [M + Na2-H]+ PA 32:4; [M + Na2-H]+ PA 36:4; [M + Na2-H]+ PC 30:1; [M + H]+ PS 24:4; [M + Na]+ PS 27:2; [M + H]+ PS 30:3; [M + Na]+ PS 37:2; [M + H]+ Plasmenyl-PC 25:0; [M + Na]+ Plasmenyl-PC 29:0; [M + Na]+ Plasmenyl-PE 34:2; [M + Na]+ Plasmenyl-PE 35:0; [M + Na]+ Plasmenyl-PE 38:5; [M + H]+ Plasmenyl-PE 42:1; [M + Na]+

Cer: Glycosphingolipids, Ceramides; CerP: Glycosphingolipids, Ceramides phosphate; DG: Neutral glycerolipid, diglyceride; LPE: P-Ethanol Amine, lysophosphatidylethanolamine; MG: Neutral glycerolipid, monoglyceride; PA: P-Acid, phosphatidic acid; PC: P-Choline, phosphatidylcholine; PS: P-Serine, phosphatidylserine; SM: Sphingolipids, sphingomyelin; TG: Neutral glycerolipid, triglyceride.

## 4. Discussion

This was an explorative study using an untargeted approach which sought to identify differences in the lipid profiles of the skin surface between WHWTs with and without AD, and between affected and unaffected skin. We detected 392 lipids, the majority of which could not be readily identified. The lipids found included ceramides, which were different to those that have been previously identified as being important in canine SC and were in relatively low abundance (Shimada et al. 2009; Yoon et al. 2011; Angelback-Schulze et al. 2014; Chermprapai et al. 2018). This suggests that there are many more lipids which may have a functional role in health and/or disease than previously identified, and the canine stratum corneum lipidome may be different to that in humans, which limits lipid identification using existing lipid databases. Alternatively, it is possible that WHWTs produce skin lipids that are significantly different to previously investigated breeds (Shimada et al. 2009; Yoon et al. 2011; Angelback-Schulze et al. 2014; Chermprapai et al. 2018). Contrary to our expectations, there was no consistent pattern that distinguished the lipid profiles in WHWTs with and without AD. Instead, the variation in lipid profiles between individual dogs was much larger than the differences between the two disease states. These findings are common in large ‘-omics’ datasets—as in this study—where frequently there are large differences between subjects and only small differences due to disease status (Westerhuis et al. 2008). It is much more statistically powerful in these situations to only analyse differences within paired measurements on the same subject so that the subject variability does not obscure the effect of the disease status (Westerhuis et al. 2008). For this reason, we utilised the statistical modelling method sPLS-DA to look at the difference in lipid profiles between affected and unaffected sites in each dog with a paired sample. Using this approach there was a clear difference between the affected and unaffected sites (Figure 4), and there were specific lipids that were reliably different in the profiles. This finding indicates that there are specific lipids that differ between affected and unaffected skin of WHWTs, which supports the secondary hypothesis of this study, and the difference was irrespective of the disease status.

The difference between affected and unaffected sites was explained predominantly by variations in the abundance of 7 unidentified lipids (X267, X135, X176, X175, X22, X48, X382) (Figure 5). Lipids X267, X135, X176 and X175 were in high abundance in unaffected sites, and in low abundance in affected sites. This was also true for lipid X22, but the relationship was weaker. Lipid X48 was more abundant in affected sites and in low abundance in unaffected sites, as was lipid X382, though less reliably.

It is possible that these are lipids produced by the sebaceous glands or corneocytes, but they could also be structural cellular lipids, or even microbially derived lipids or degradation products. It was beyond the scope of this explorative study to identify lipids that did not match with the published databases and it is clear that further chemical characterisation will be required. Given that some or all of them could be pathophysiologically relevant, further targeted lipidomic techniques are indicated to characterise and quantify these lipids. To facilitate further research, we have provided the full data set (Supporting information 3) and it has also been registered with the Open Science Framework (https://osf.io/7v9yk/).

There were several limitations to this study including those inherent to a relatively small group of dogs and it was difficult to find WHWTs that met the strict non-atopic criteria implemented. However, given the individual variation, it is unlikely that there would have been separation between atopic and non-atopic, even with a larger number of dogs. A paired sample was not collected from every dog (unpaired sample for 4/7 HNA dogs) which was a limitation for the statistical analysis. There were also only 5/39 dogs under 12 months of age, and the groups were not age or gender matched. It is not known how these variables may have impacted the lipid profiles obtained but it is known from human studies that skin lipids vary with age and gender (Ghadially et al. 1995; Rogers et al. 1996; Szöllősi et al. 2017). Lipid profiles also vary between body sites (Chermprapai et al. 2018) and, in an untargeted human study, lipids varied depending on sebaceous gland density (Ludovici et al. 2018). To limit this variable in our study the body sites sampled were kept as consistent as possible but there was variation in the affected sites sampled, and it is not known if this may have influenced the results. There were only 2/7 HNA dogs under 18 months of age and it is possible that these dogs may yet develop AD. We only studied the skin lipids of one breed of dog and the study only included WHWT from New Zealand which has limitations and advantages. An advantage was that breed was not a variable, however the absence of a difference between HNA and AD dogs could be attributed to an inherent idiosyncrasy in the lipids of the skin of the WHWT. Comparison of skin surface lipids of different breeds of dogs is an area of future study. In addition, as the skin surface lipids have previously been ignored, it is unknown what sampling approach gives the best diagnostic and research information. Another limitation to the study was the variation in several other factors, including topical and systemic medication, shampoo usage and diet, all of which can affect cutaneous lipid content. This was an explorative study and as such this variation was expected and was at least partially accounted for with the statistical analysis methods utilised.

## 5. Conclusions

Investigation into the lipidome of the skin surface in health and disease is an emerging area of research which could have clinical and therapeutic applications. In this study, an untargeted approach revealed a vast array of lipids present on the surface of the skin in WHWTs, the majority of which could not be identified. There was no overall pattern in the skin lipid profile that distinguished between WHWTs with and without AD. However, there was a pattern that distinguished affected from unaffected skin and this was irrespective of the disease status of the dog. This difference may be due to consistent changes to the skin lipids induced by inflammation or infection however it remains to be seen what the key pathophysiological changes are, and the identity and functional significance of the lipids associated with this difference.

## Data Availability

All data generated or analysed during this study are included in this published article and the Additional and supporting information files and full lipid data set is available on the Open Science Framework (https://osf.io/7v9yk/).
